# The role of hybrid ubiquitin chains in the MyD88 and other innate immune signalling pathways

**DOI:** 10.1038/cdd.2017.17

**Published:** 2017-05-05

**Authors:** Philip Cohen, Sam Strickson

**Affiliations:** 1 MRC Protein Phosphorylation and Ubiquitylation Unit, School of Life Sciences, University of Dundee, Dundee DD1 5EH, UK

## Abstract

The adaptor protein MyD88 is required for signal transmission by toll-like receptors and receptors of the interleukin-1 family of cytokines. MyD88 signalling triggers the formation of Lys63-linked and Met1-linked ubiquitin (K63-Ub, M1-Ub) chains within minutes. The K63-Ub chains, which are formed by the E3 ubiquitin ligases TRAF6, Pellino1 and Pellino2, activate TAK1, the master kinase that switches on mitogen-activated protein (MAP) kinase cascades and initiates activation of the canonical I*κ*B kinase (IKK) complex. The M1-Ub chains, which are formed by the linear ubiquitin chain assembly complex (LUBAC), bind to the NEMO (NF-*κ*B essential modulator) component of the IKK complex and are required for TAK1 to activate IKKs, but not MAP kinases. An essential E3 ligase-independent role of TRAF6 is to recruit LUBAC into the MyD88 signalling complex, where it recognises preformed K63-Ub chains attached to protein components of these complexes, such as IRAK1 (IL-1 receptor-associated kinase), producing ubiquitin chains containing both types of linkage, termed K63/M1-Ub hybrids. The formation of K63/M1-Ub hybrids, which is a feature of several innate immune signalling pathways, permits the co-recruitment of proteins that interact with either K63-Ub or M1-Ub chains. Two likely roles for K63/M1-Ub hybrids are to facilitate the TAK1-dependent activation of the IKK complex and to prevent the hyperactivation of these kinases by recruiting A20 and A20-binding inhibitor of NF-*κ*B1 (ABIN1). These proteins restrict activation of the TAK1 and IKK complexes, probably by competing with them for binding to K63/M1-Ub hybrids. The formation of K63/M1-Ub hybrids may also regulate the rate at which the ubiquitin linkages in these chains are hydrolysed. The IKK-catalysed phosphorylation of some of its substrates permits their recognition by the E3 ligase SCF^*β*TRCP^, leading to their Lys48-linked ubiquitylation and proteasomal degradation. Innate immune signalling is therefore controlled by the formation and destruction of three different types of ubiquitin linkage.

## Facts

- The activation of toll-like receptors (TLR) and the interleukin-1 (IL-1) receptor triggers the rapid formation of Lys63-linked and Met1-linked ubiquitin oligomers.
- The Lys63-linked and Met1-linked ubiquitin oligomers are required to activate the TAK1 and canonical I*κ*B kinase complexes, respectively.
- The Met1-linked ubiquitin oligomers are attached covalently to preformed Lys63-linked ubiquitin oligomers, generating Lys63/Met1 ubiquitin hybrids.
- The Lys63/Met1 ubiquitin hybrids can recruit proteins that bind to either Lys63-linked or Met1-linked ubiquitin oligomers.

## Open Questions

- Which E3 ligases generate the Lys63-linked ubiquitin chains that are required for the activation of TAK1 and the formation of Lys63/Met1 ubiquitin hybrids?
- In the TLR and IL-1R signalling pathways, how is LUBAC (linear ubiquitin chain assembly complex) able to use Lys63-linked ubiquitin oligomers as its preferred initial substrate, rather than monomeric ubiquitin?
- How is the TLR- or IL-1R-dependent formation of Met1-linked ubiquitin chains regulated?
- What roles do Lys63/Met1-linked ubiquitin hybrids play in innate immune signalling pathways?

## Lys63-Linked Ubiquitin Oligomers Activate the Protein Kinase TAK1

The role of Lys48-linked ubiquitin (K48-Ub) oligomers in triggering protein degradation by the proteasome was worked out between 1978 and 1989,^1, 2^ but it was not until the mid 1990s that cellular roles for other types of Ub oligomers began to emerge. In 1995, yeast expressing the Ub[K63R] mutation were shown to be hypersensitive to ultraviolet irradiation and failed to make a particular subset of ubiquitin conjugates.^3^ This provided indirect evidence that K63-Ub oligomers were involved in DNA repair, but direct evidence was only obtained 4 years later when Ubc13, a protein known to be required for DNA repair, was shown to assemble K63-Ub chains specifically in the presence of yeast orthologues of the mammalian protein Uev1a.^4^ The same paper also provided genetic evidence that the enhanced UV sensitivity of yeast expressing Ubc13 mutants was caused by the failure to synthesise these ubiquitin chains.

Soon after, tumour necrosis factor receptor-associated factor 6 (TRAF6), a protein whose expression is essential for interleukin-1 (IL-1) and toll-like receptor (TLR) signalling,^5, 6, 7^ was shown to possess E3 ubiquitin ligase activity, which in combination with the Ubc13-Uev1a E2-conjugating complex catalysed the specific formation of Lys63-linked ubiquitin (K63-Ub) chains.^8^ Even more importantly, the TRAF6-generated K63-Ub chains were found to activate a protein kinase *in vitro*, which could in turn activate the canonical I*κ*B kinase (IKK) complex and MAP kinase kinase 6 (MKK6) *in vitro*. It was purified and identified as the TAK1 (transforming growth *β*-activated kinase 1) complex, comprising the TAK1 catalytic subunit, TAK1-binding protein 1 (TAB1) and TAB2.^9^ Subsequently TAB3, a TAB2-related protein, was identified^10^ and it became clear that two TAK1 complexes (TAB1–TAK1–TAB2 and TAB1–TAK1–TAB3) are expressed in cells.^11^ The essential role of TAK1 in mediating the IL-1-dependent activation of the canonical IKK complex and MAP kinases was demonstrated by studies in embryonic fibroblasts from TAK1 knockout (KO) mice^12^ or knock-in mice lacking TAK1 catalytic activity.^13^

The C-termini of TAB2 and TAB3 possess Npl4 zinc fingers (NZF) that bind to K63-Ub chains specifically and are essential for the activation of TAK1 *in vitro*.^14, 15^ Thus the idea emerged that TRAF6-generated K63-Ub chains interact with TAB2 or TAB3 inducing a conformational change that activates the TAK1 catalytic subunit. This allosteric mechanism is similar to the activation of cyclic AMP-dependent protein kinase (PKA) by cyclic AMP or calmodulin-dependent protein kinases by calcium ions.

## How MyD88 Signalling Activates the E3 Ligases TRAF6 and Pellino

**Figure 1:**
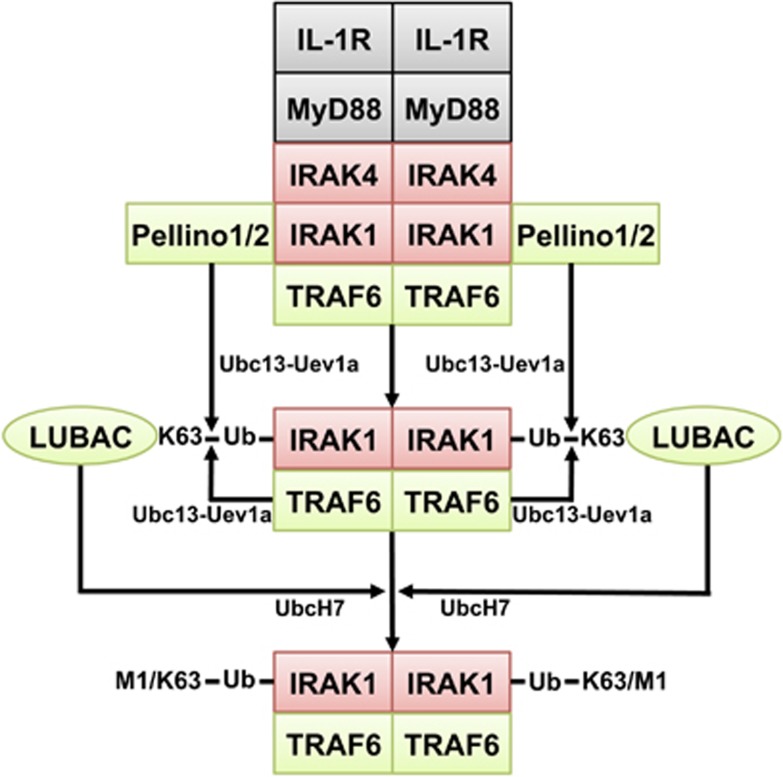
How the MyD88 pathway triggers the ubiquitylation of IRAK1. The interaction of IL-1 with its receptor leads to the formation of the Myddosome and the phosphorylation and activation of IRAK1. The interaction of IRAK1 with TRAF6 induces dimerisation of the RING domain of TRAF6, activating its E3 ligase function. IRAK1 also phosphorylates Pellino1 and Pellino2 converting them from inactive to active E3 ubiquitin ligases. In the presence of E1-activating enzyme and the E2-conjugating complex Ubc13-Uev1a, TRAF6 and Pellinos 1 and 2 both contribute to the formation of K63-Ub chains, which become attached to IRAK1 and other components of the Myddosome. One essential role of TRAF6 is to recruit LUBAC into the IL-1 signalling complex where it interacts with K63-Ub-substrates, such as IRAK1, to produce K63/M1-Ub-IRAK1. In the human cell lines we have studied, the expression of IRAK1 is essential for IL-1 signalling and the IRAK2 in these cells cannot compensate for the loss of IRAK1. Protein kinases are highlighted in red and E3 ligases in green

Myeloid differentiation primary response gene 88 (MyD88) is an adaptor protein required for signal transduction by TLRs and receptors of the IL-1 family of cytokines (IL-1, IL-18 and IL-33). The interaction of components of microbial pathogens with TLRs or IL-1 with the IL-1R recruits MyD88 to the receptors, and then members of the IL-1 receptor-associated kinase (IRAK) family to MyD88 through a series of death domain (DD) interactions. The DD of IRAK4 interacts with the DD of MyD88 and the DDs of IRAK1 and/or IRAK2 with the DD of IRAK4, forming oligomeric complexes termed Myddosomes.^16, 17^ TRAF6 interacts with Pro-Xaa-Glu motifs in the C-terminal regions of IRAK1 and IRAK2, triggering the dimerisation of the RING domain of TRAF6^18^ and the activation of its E3 ligase activity (Figure 1).^19^ In contrast to TRAF6, the Pellino family of E3 ligases is activated by IRAK-catalysed phosphorylation. They can be activated *in vitro* by IRAK1 or IRAK4^20, 21^ and the IL-1-dependent activation of Pellino1 is suppressed in embryonic fibroblasts from knock-in mice expressing a kinase-inactive mutant of IRAK1 or in human cells treated with an IRAK1 inhibitor.^22^ Like TRAF6, the Pellino E3 ligases can combine with Ubc13-Uev1a to generate K63-Ub chains *in vitro*.^20, 21^

It is widely accepted that the E3 ligase activity of TRAF6 is essential for MyD88 signalling. However, we have found that E3 ligase-inactive mutants of TRAF6 partially restore IL-1 and TLR signalling when they are re-expressed in TRAF6 KO human cells, but not in TRAF6 KO cells that also lack expression of Pellino1 and Pellino2. Moreover, we have generated a knock-in mouse in which TRAF6 is replaced by an E3 ligase-inactive mutant, and shown that MyD88 signalling is reduced, but not abolished in primary macrophages and embryonic fibroblasts from these mice.^23^ These and other findings indicate that TRAF6, Pellino1 and Pellino2 contribute to the MyD88-dependent formation of K63-Ub chains in some cells (Figure 1), and that the essential roles of TRAF6 in this pathway are independent of its E3 ligase activity.

## Met1-Linked Ubiquitin Oligomers and the MyD88-Dependent Activation of the Canonical IKK Complex

The canonical IKK complex comprises the related protein kinases IKK*α* and IKK*β,* and a regulatory component called NF-*κ*B essential modulator (NEMO).^24, 25^ NEMO possesses an ubiquitin-binding domain that was initially shown to interact with K63-Ub oligomers.^26, 27^ Mutations in this domain, such as NEMO[D311N], prevent binding to ubiquitin oligomers, impair activation of the canonical IKK complex and cause X-linked recessive anhidrotic ectodermal dysplasia with immunodeficiency in humans.^28, 29^ These findings highlighted the importance of ubiquitin binding to NEMO for the activation of the canonical IKK complex *in vivo*. They also suggested that K63-Ub chains might function as molecular scaffolds to co-recruit the IKK and TAK1 complexes, thereby facilitating the TAK1-dependent activation of the IKKs.^26^

**Figure 2:**
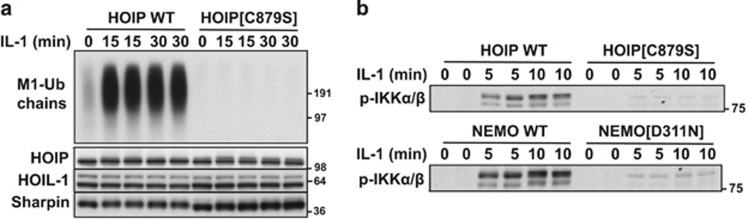
The formation of M1-Ub chains and their interaction with NEMO are required for TAK1 to initiate the activation of the canonical IKK complex. (**a**) IL-1 α induces the formation of M1-Ub chains in embryonic fibroblasts from wild-type (WT) mice, but not in knock-in mice expressing the E3 ligase-inactive HOIP[C879S] mutant. (**b**) The IL-1α-dependent, TAK1-catalysed phosphorylation of IKK*β* at Ser177 is suppressed in embryonic fibroblasts from HOIP[C879S] knock-in mice that do not form M1-Ub chains, or in embryonic fibroblasts from NEMO[D311N] knock-in mice in which M1-Ub chains are formed, but are unable to interact with NEMO. Adapted from Emmerich *et al.*^36^ and Zhang *et al.*^37^

An unexpected twist to the regulation of the IKK complex emerged with the discovery of the linear ubiquitin chain assembly complex (LUBAC), a heterotrimeric complex comprising HOIP (the catalytic subunit), HOIL-1 and Sharpin.^30, 31, 32, 33^ LUBAC is a unique E3 ubiquitin ligase, which catalyses the formation of Met1-linked ubiquitin (M1-Ub) chains (also called linear ubiquitin chains) in which the N-terminal methionine of one ubiquitin forms a peptide bond with the C-terminal carboxylate of another ubiquitin molecule. Significantly, NEMO was found to bind to M1-Ub dimers with 100-fold higher affinity than K63-Ub dimers.^34, 35^ Moreover, the IL-1-dependent formation of M1-Ub chains was abolished (Figure 2a) and activation of the canonical IKK complex greatly reduced (Figure 2b) in embryonic fibroblasts from knock-in mice in which HOIP had been replaced by the E3 ligase-inactive HOIP[C879S] mutant.^36^ The IL-1-dependent activation of the canonical IKK complex was similarly reduced in embryonic fibroblasts from knock-in mice expressing the NEMO[D311N] mutant^37^ (Figure 2b). Thus the LUBAC-catalysed formation of M1-Ub chains and their binding to NEMO are both required for robust IL-1-dependent activation of the canonical IKK complex in murine embryonic fibroblasts (MEFs). The IL-1-dependent activation of the IKK complex is also impaired in embryonic fibroblasts from HOIL-1 KO mice, which may be explained by the greatly reduced expression of HOIP in these cells.^38^ Consistent with the genetic evidence outlined above, LUBAC-generated M1-Ub chains restored the activation of the canonical IKK complex in HeLa cell extracts depleted of these components, while the overexpression of LUBAC in wild-type MEFs, but not in NEMO-deficient MEFs, induced NF-*κ*B-dependent gene transcription.^39^

The activation of IKK*α* and IKK*β* requires the phosphorylation of two amino-acid residues in the activation loops of these protein kinases (Ser176 and Ser180 of IKK*α*; Ser177 and Ser181 of IKK*β*).^40^ More recently the activation of IKK*β* was shown to take place by a two-step mechanism in which TAK1 first phosphorylates IKK*β* at Ser177, which is followed by the IKK*β*-catalysed auto-phosphorylation of Ser181.^37^ The IL-1-dependent phosphorylation of IKK*β* at Ser177 (or IKK*α* at Ser176) is impaired in embryonic fibroblasts from the HOIP[C879S] or NEMO[D311N] mice (Figure 2b). Thus, the formation of M1-Ub chains and their interaction with NEMO are both needed for TAK1 to initiate activation of the IKK complex.^37^ The interaction of M1-Ub chains with NEMO may induce a conformational change that permits TAK1 to phosphorylate Ser176/177 of IKK*α*/IKK*β*.

The formation of M1-Ub chains is not required for the IL-1-dependent activation of p38*α* MAP kinase, c-Jun N-terminal kinase 1 (JNK1) and JNK2.^36^

## The Role of Lys48-Linked Ubiquitylation in MyD88 Signalling

**Figure 3:**
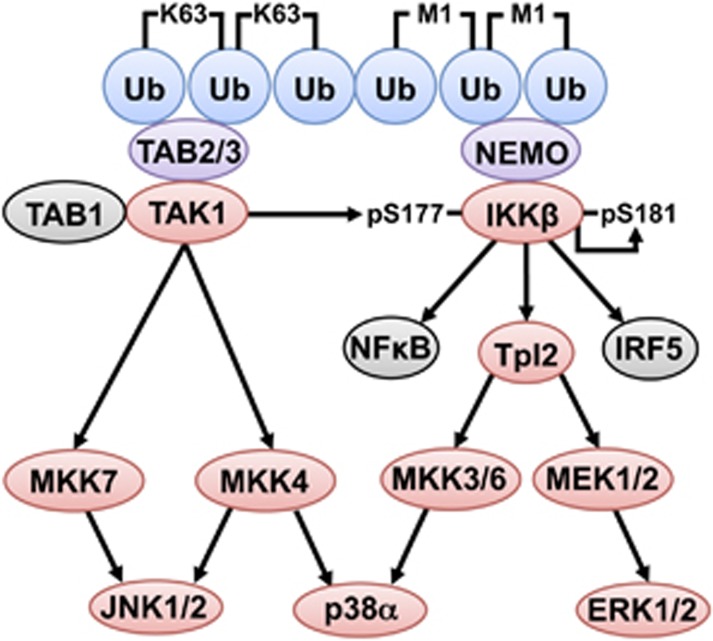
The formation of K63/M1-Ub hybrids facilitates the activation of TAK1 and IKK complexes, and the phosphorylation of their substrates. The K63/M1-Ub hybrids produced by innate immune signalling pathways recruit the TAK1 and IKK complexes. The interaction of K63-Ub chains with TAB2 and TAB3 activates TAK1 allowing it to phosphorylate and activate MAP kinase kinases 4 and 7 (MKK4, MKK7), which activate the MAP kinases, termed JNK1 and JNK2. The binding of M1-Ub chains to NEMO induces a conformational change that permits TAK1 to phosphorylate IKK*β* at Ser177. IKK*β* completes the activation process by phosphorylating itself at Ser181. IKK*β* can then activate transcription factors essential for inflammatory mediator production (NF-*κ*B and IRF5). IKK*β* also activates the Tpl2 kinase complex, which activates MEK1 and MEK2, the protein kinases that activate ERK1 and ERK2. Tpl2 additionally phosphorylates MKK3 and MKK6. MKK3 and MKK6 operate redundantly with MKK4 to phosphorylate and activate p38*α* in some cells. Protein kinases are highlighted in red, ubiquitin-binding proteins in purple and ubiquitin (Ub) molecules in blue. Further details are given in the text

IKK*β* activates the transcription factor NF-*κ*B by phosphorylating its inhibitory (I*κ*B) components. For example, the dual phosphorylation of I*κ*B*α* at Ser32 and Ser36 recruits the SCF*β*^TRCP^ E3 ligase, leading to Lys48-linked ubiquitylation and proteasomal degradation of IκBα. The p65 and p50 components of NF-*κ*B can then translocate to the nucleus as a heterodimer to stimulate the transcription of NF-*κ*B-dependent genes. IKK*β* also phosphorylates the transcription factor IRF5 (interferon regulatory factor 5) at Ser462, inducing its dimerisation and nuclear translocation where it switches on the transcription of genes encoding major inflammatory cytokines, such as IL-12^41^ and interferon *β*.^42, 43^ Thus IKK*β* switches on two of the ‘master’ transcription factors required for inflammatory mediator production (Figure 3).

Another important substrate of IKK*β* is the protein kinase Tpl2 (tumour promotion locus 2). Two heterotrimeric Tpl2 complexes are expressed in cells, comprising the Tpl2_L_ or Tpl2_S_ catalytic subunits, p105 (also called NF-*κ*B1, the precursor of the p50 component of NF-*κ*B) and ABIN2 (A20-binding inhibitor of NF-*κ*B2).^44^ Tpl2_L_ and Tpl2_S_ are products of the same gene, and arise from the use of alternative initiation start sites at Met1 (Tpl2_L_) or Met30 (Tpl2_S_).^45^ IKK*β* has a dual role in the activation of Tpl2. First it phosphorylates p105 at Ser927 and Ser932, leading to the SCF*β*^TRCP^-catalysed Lys48-linked ubiquitylation of p105 and its proteasomal degradation;^46, 47^ second it phosphorylates Tpl2_L_ at Ser400, which is followed by the Tpl2-catalysed phosphorylation of Ser443 and its interaction with 14-3-3 proteins. The activated Tpl2_L_ is subsequently ubiquitylated and degraded by the proteasome.^46, 47^ In contrast, Tpl2_S_ is not degraded and is presumably responsible for maintaining Tpl2 activity at a lower level for the prolonged period (4–8 h) needed to produce substantial amounts of pro-inflammatory cytokines in myeloid cells.

The substrates of Tpl2 identified so far are MKK family members, namely MAP kinase or ERK kinase 1 (MEK1), MEK2, MKK3 and MKK6 (Figure 3). MEK1 and MEK2 activate ERK1 and ERK2 (extracellular signal-regulated kinases 1 and 2).^48^ MKK3 and MKK6 are rate limiting for the activation of p38*α* MAP kinase in some cells, but not in others cells where MKK4 can activate p38*α* MAP kinase if MKK3 and MKK6 are not activated^49^ (Figure 3). ERK1/2 and p38*α* MAP kinase phosphorylate many proteins that control the transcription, translation, processing and secretion of inflammatory mediators.^50^

Interestingly, the ABIN2 component of Tpl2 complexes possesses a ubiquitin-binding domain similar to that present in NEMO,^51^ but how and whether the binding of ubiquitin chains to ABIN2 regulates Tpl2 activity is unknown.

## How is LUBAC Regulated?

The activation of the MyD88 signalling pathway triggers the formation of M1-Ub chains within minutes, but the intrinsic E3 ligase activity of LUBAC is not increased.^36^ These observations indicate that LUBAC is not converted from an inactive to an active form by a stable covalent modification, such as phosphorylation. Instead, as discussed later, the formation of M1-Ub chains is likely to be triggered by the recruitment of LUBAC into the MyD88 signalling complex where it is targeted to its substrates by TRAF6. However, the formation of M1-Ub chains may be facilitated by the inhibition of Otulin and/or CYLD (the product of the Cylindromatosis gene), which appear to be the major deubiquitylases that hydrolyse M1-Ub chains, as discussed elsewhere in this issue.^52^ Interestingly, Otulin binds to the PUB (N-terminal peptide: N-glycanase/UBA- or UBX-containing protein) domain of HOIP, while CYLD is also recruited to the PUB domain of HOIP, but indirectly via the protein SPATA2 (spermatogenesis-associated protein 2).^52^ The formation and destruction of M1-Ub chains is therefore catalysed by a single multi-enzyme complex.

## Hybrid Ubiquitin Chains Containing Both M1-Ub and K63-Ub Linkages

Most of the M1-Ub chains formed in response to IL-1*β* or the TLR1/2 agonist Pam_3_CSK_4_ are attached covalently to K63-Ub chains, forming ubiquitin chains containing both types of linkage, hereafter called K63/M1-Ub ‘hybrids’.^36^ Some of the K63/M1-Ub hybrids present in cell extracts are ‘anchored’ covalently to the components of the Myddosome, and most strikingly to IRAK1 (Figure 1). However, K63/M1-Ub hybrids that are not ‘anchored’ covalently to any other protein can also be detected in extracts prepared from IL-1-stimulated cells.^36^ Whether these ‘unanchored’ K63/M1-Ub hybrids are formed *de novo* or generated by cleavage from ‘anchored’ K63/M1-Ub hybrids is unknown. The relative importance of anchored and unanchored K63/M1-Ub hybrids in activating the TAK1 and IKK complexes remains to be clarified.

When the M1-Ub chains present in cell extracts are hydrolysed by incubation with Otulin, the size of the Ub chains attached to IRAK1 is reduced, but no mono-ubiquitylated IRAK1 is generated. This finding, together with the reduced formation of M1-Ub chains in Ubc13-deficient cells, indicates that IRAK1 is first modified by Lys63-linked ubiquitylation, the M1-Ub linkages being added subsequently.^36^ Therefore, although LUBAC can assemble M1-Ub chains from monomeric ubiquitin *in vitro*, it is K63-Ub oligomers and not monomeric ubiquitin, which are the substrates first used by LUBAC when it is recruited into the MyD88 signalling complex.^36^ Interestingly, the MyD88-dependent formation of M1-Ub chains is drastically reduced in TRAF6 KO cells, and can be restored by the re-expression of wild-type or E3 ligase-inactive mutants of TRAF6. The specific recognition of K63-Ub-substrates by LUBAC may be facilitated by the NZF domains present in HOIP, which interact specifically with K63-Ub chains *in vitro*.^36, 53^

The specific hydrolysis of the M1-Ub chains present in K63/M1-Ub hybrids liberates small K63-Ub oligomers of varying length and, conversely, the specific hydrolysis of K63-Ub chains liberates small M1-Ub oligomers of varying length.^36^ These experiments demonstrate that the topology of the K63/M1-Ub hybrids is complex. They also indicate that once the first M1-Ub linkage has been made to a preformed K63-Ub oligomer, LUBAC can then use monomeric ubiquitin as a substrate to add additional M1-Ub linkages. The HOIL-1 component of LUBAC also possesses NZF domains but, in contrast to those present in HOIP, they bind to M1-Ub oligomers more strongly than to K63-Ub oligomers.^36, 54^ This could operate as a positive feedback loop to recruit more molecules of LUBAC to the MyD88 signalling complex once the initial M1-Ub linkages have been formed.

## Potential Roles of the K63/M1-Ub Hybrids

The formation of hybrid ubiquitin chains containing both K63-Ub and M1-Ub linkages is not confined to the MyD88 signalling network, but is a feature of at least three other innate immune signalling pathways. These include the nucleotide oligomerisation domain 1 (NOD1) and NOD2 pathways that are activated by components of bacterial peptidoglycans, the TLR3 pathway, which responds to viral double-stranded RNA and signals via the adaptor protein TRIF (TIR domain-containing adapter-inducing interferon-*β*), and the TNF (tumour necrosis factor) signalling pathway.^55, 56^ These observations suggest that the formation of K63/M1-Ub hybrids is of general significance for the regulation of these signal transduction pathways, raising the question of what their specific roles might be.

The MyD88, TRIF, NOD1/2 and TNF signalling pathways all lead to the rapid, TAK1-dependent activation of the canonical IKK complex. Since the TAB2 and TAB3 components of TAK1 complexes interact specifically with K63-Ub oligomers, and the NEMO component of the canonical IKK complex binds selectively to M1-Ub oligomers, the formation of K63/M1-Ub hybrids may permit the co-recruitment of these two kinase complexes to the same ubiquitin chains (Figure 3). One role for the K63/M1-Ub hybrids may therefore be to facilitate the TAK1-dependent activation of the IKK complex.^37^

**Figure 4:**
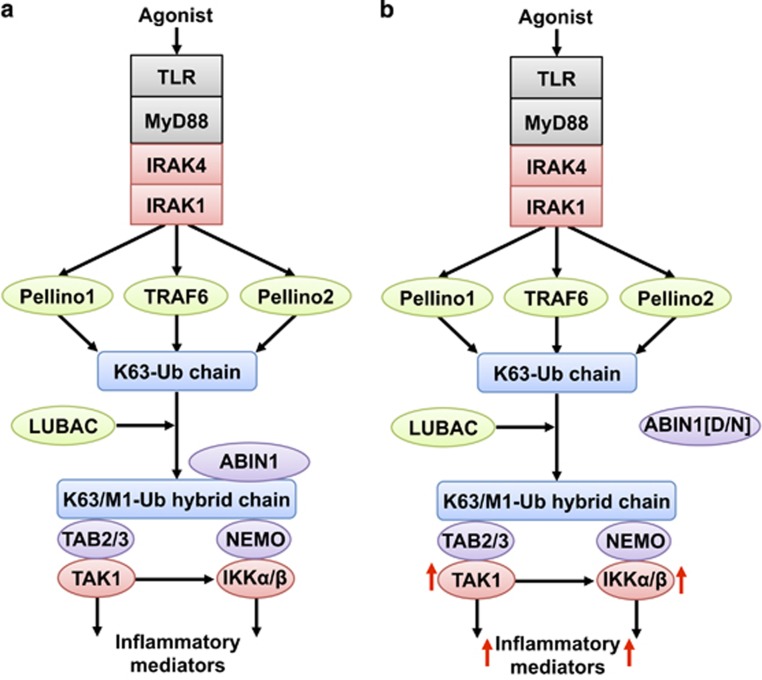
ABIN1 restricts the activation of TAK1 and the canonical IKK complex. (**a**) ABIN1 binds to K63/M1-Ub hybrids and competes with the TAK1 and IKK complexes for binding to these ubiquitin chains. This suppresses the interaction of the TAK1 and IKK complexes with these ubiquitin chains, restricting their activation and the production of inflammatory mediators. (**b**) In dendritic cells and B cells from knock-in mice expressing the ubiquitin-binding-defective ABIN1[D485N] mutant, this restriction is removed, leading to hyperactivation of the TAK1 and IKK complexes and the overproduction of inflammatory mediators (red arrows). This leads to the spontaneous development of SLE when the ABIN1[D485N] mice are 4–5 months old. The colour key is as in Figures 1 and 3

However, K63/M1-Ub hybrids are likely to control the activation of innate immune signalling in other ways. For example, the protein ABIN1 possesses a ubiquitin-binding domain similar to that present in NEMO.^51^ ABIN1 restricts the activation of TAK1 and the canonical IKK complex, probably by competing with TAB2/3 and NEMO for binding to K63/M1-Ub hybrids (Figure 4a). The genetic evidence supporting this hypothesis comes from studies with mice in which the gene encoding ABIN1 was replaced by the ubiquitin-binding-defective ABIN1[D485N] mutant. This variant is equivalent to the NEMO[D311N] mutation discussed earlier. The MyD88-dependent activation of TAK1, IKKs and MAPKs, and the production of inflammatory mediators, was enhanced several fold in dendritic cells and B cells from the ABIN1[D485N] mice^57^ (Figure 4b). Interestingly, the ABIN1[D485N] knock-in mice spontaneously developed an autoimmune disease similar to Type III and Type IV systemic lupus erythematosus (SLE) in humans, which can be prevented by crossing the ABIN1[D485N] mice to MyD88 KO mice.^57^ This demonstrates that enhanced MyD88 signalling causes the disease. SLE was also prevented by crossing ABIN1[D485N] mice to knock-in mice expressing kinase-inactive mutants of IRAK1 or IRAK4.^58^ ABIN1 polymorphisms have been shown to predispose to lupus, psoriasis and other autoimmune diseases in eight human populations (see Nanda *et al.*^58^ for more information), suggesting that drugs inhibiting IRAK1 and/or IRAK4 could have therapeutic potential for the prevention and perhaps the treatment of lupus caused by hyperactivation of the MyD88 signalling network.

The ABIN1-interacting protein A20 is an immediate early gene whose expression is increased when the MyD88 signalling pathway is activated, and which also prevents the overproduction of inflammatory mediators by this pathway.^52^ The C-terminal domain of A20 contains seven zinc fingers. The fourth and seventh zinc fingers bind to K63-Ub^59, 60^ and M1-Ub oligomers,^61, 62^ respectively, making A20 a prime candidate to interact with K63/M1-Ub hybrids and compete with the TAK1 and IKK complexes for binding to these molecules. Since ABIN1 and A20 both interact with K63/M1-Ub hybrids and with each other,^63^ ternary complexes formed between ABIN1, A20 and K63/M1-Ub hybrids may restrict the activation of the TAK1 and IKK complexes more effectively than either ABIN1 or A20 alone (Figure 4). These observations can explain why A20 has been described as a ‘central gatekeeper’ in inflammation and immunity^64^ and it also prevents some types of lymphoma.^65^

The N-terminal domain of A20 possesses deubiquitylase activity. It was recently reported that the covalent attachment of M1-Ub oligomers to K63-Ub oligomers reduces the rate at which A20 can hydrolyse K63-Ub linkages *in vitro*, suggesting a novel role for K63/M1-Ub hybrids in regulating the rate of Ub chain hydrolysis.^66^ It was also reported that the IKK*β*-catalysed phosphorylation of A20 enhances the rate at which A20 hydrolyses K63-Ub chains.^66^ However, the physiological significance of the A20 deubiquitylase activity merits further attention, because other investigators reported that knock-in mice expressing a deubiquitylase-inactive mutant of A20 appeared normal, with no signs of inflammation. The mice also had normal proportions of B, T, dendritic and myeloid cells. Moreover lipolysaccharide and TNF responses, such as NF-*κ*B activation, were unaffected.^67^

## References

[bib1] Chau V, Tobias JW, Bachmair A, Marriott D, Ecker DJ, Gonda DK et al. A multiubiquitin chain is confined to specific lysine in a targeted short-lived protein. Science 1989; 243: 1576–1583. 2538923 10.1126/science.2538923

[bib2] Ciehanover A, Hod Y, Hershko A. A heat-stable polypeptide component of an ATP-dependent proteolytic system from reticulocytes. Biochem Biophys Res Commun 1978; 81: 1100–1105. 666810 10.1016/0006-291x(78)91249-4

[bib3] Spence J, Sadis S, Haas AL, Finley D. A ubiquitin mutant with specific defects in DNA repair and multiubiquitination. Mol Cell Biol 1995; 15: 1265–1273. 7862120 10.1128/mcb.15.3.1265 PMC230349

[bib4] Hofmann RM, Pickart CM. Noncanonical MMS2-encoded ubiquitin-conjugating enzyme functions in assembly of novel polyubiquitin chains for DNA repair. Cell 1999; 96: 645–653. 10089880 10.1016/s0092-8674(00)80575-9

[bib5] Cao Z, Xiong J, Takeuchi M, Kurama T, Goeddel DV. TRAF6 is a signal transducer for interleukin-1. Nature 1996; 383: 443–446. 8837778 10.1038/383443a0

[bib6] Lomaga MA, Yeh W-C, Sarosi I, Duncan GS, Furlonger C, Ho A et al. TRAF6 deficiency results in osteopetrosis and defective interleukin-1, CD40, and LPS signaling. Genes Dev 1999; 13: 1015–1024. 10215628 10.1101/gad.13.8.1015 PMC316636

[bib7] Naito A, Azuma S, Tanaka S, Miyazaki T, Takaki S, Takatsu K et al. Severe osteopetrosis, defective interleukin-1 signalling and lymph node organogenesis in TRAF6-deficient mice. Genes Cells 1999; 4: 353–362. 10421844 10.1046/j.1365-2443.1999.00265.x

[bib8] Deng L, Wang C, Spencer E, Yang L, Braun A, You J et al. Activation of the IκB kinase complex by TRAF6 requires a dimeric ubiquitin-conjugating enzyme complex and a unique polyubiquitin chain. Cell 2000; 103: 351–361. 11057907 10.1016/s0092-8674(00)00126-4

[bib9] Wang C, Deng L, Hong M, Akkaraju GR, Inoue J, Chen ZJ. TAK1 is a ubiquitin-dependent kinase of MKK and IKK. Nature 2001; 412: 346–351. 11460167 10.1038/35085597

[bib10] Ishitani T, Takaesu G, Ninomiya-Tsuji J, Shibuya H, Gaynor RB, Matsumoto K. Role of the TAB2-related protein TAB3 in IL-1 and TNF signaling. EMBO J 2003; 22: 6277–6288. 14633987 10.1093/emboj/cdg605 PMC291846

[bib11] Cheung PCF, Nebreda AR, Cohen P. TAB3, a new binding partner of the protein kinase TAK1. Biochem J 2004; 378: 27–34. 14670075 10.1042/BJ20031794 PMC1223947

[bib12] Shim J-H, Xiao C, Paschal AE, Bailey ST, Rao P, Hayden MS et al. TAK1, but not TAB1 or TAB2, plays an essential role in multiple signaling pathways *in vivo*. Genes Dev 2005; 19: 2668–2681. 16260493 10.1101/gad.1360605 PMC1283960

[bib13] Sato S, Sanjo H, Takeda K, Ninomiya-Tsuji J, Yamamoto M, Kawai T et al. Essential function for the kinase TAK1 in innate and adaptive immune responses. Nat Immunol 2005; 6: 1087–1095. 16186825 10.1038/ni1255

[bib14] Kanayama A, Seth RB, Sun L, Ea C-K, Hong M, Shaito A et al. TAB2 and TAB3 activate the NF-kappaB pathway through binding to polyubiquitin chains. Mol Cell 2004; 15: 535–548. 15327770 10.1016/j.molcel.2004.08.008

[bib15] Kulathu Y, Akutsu M, Bremm A, Hofmann K, Komander D. Two-sided ubiquitin binding explains specificity of the TAB2 NZF domain. Nat Struct Mol Biol 2009; 16: 1328–1330. 19935683 10.1038/nsmb.1731

[bib16] Motshwene PG, Moncrieffe MC, Grossmann JG, Kao C, Ayaluru M, Sandercock AM et al. An oligomeric signaling platform formed by the toll-like receptor signal transducers MyD88 and IRAK-4. J Biol Chem 2009; 284: 25404–25411. 19592493 10.1074/jbc.M109.022392 PMC2757241

[bib17] Lin S-C, Lo Y-C, Wu H. Helical assembly in the MyD88-IRAK4-IRAK2 complex in TLR/IL-1R signalling. Nature 2010; 465: 885–890. 20485341 10.1038/nature09121 PMC2888693

[bib18] Ye H, Arron JR, Lamothe B, Cirilli M, Kobayashi T, Shevde NK et al. Distinct molecular mechanism for initiating TRAF6 signalling. Nature 2002; 418: 443–447. 12140561 10.1038/nature00888

[bib19] Yin Q, Lin S, Lamothe B, Lu M. E2 interaction and dimerization in the crystal structure of TRAF6. Nat Struct Mol Biol 2009; 16: 658–666. 19465916 10.1038/nsmb.1605 PMC2834951

[bib20] Smith H, Peggie M, Campbell DG, Vandermoere F, Carrick E, Cohen P. Identification of the phosphorylation sites on the E3 ubiquitin ligase Pellino that are critical for activation by IRAK1 and IRAK4. Proc Natl Acad Sci USA 2009; 106: 4584–4590. 19264966 10.1073/pnas.0900774106 PMC2651833

[bib21] Ordureau A, Smith H, Windheim M, Peggie M, Carrick E, Morrice N et al. The IRAK-catalysed activation of the E3 ligase function of Pellino isoforms induces the Lys63-linked polyubiquitination of IRAK1. Biochem J 2008; 409: 43–52. 17997719 10.1042/BJ20071365 PMC5791886

[bib22] Goh ETH, Arthur JSC, Cheung PCF, Akira S, Toth R, Cohen P. Identification of the protein kinases that activate the E3 ubiquitin ligase Pellino 1 in the innate immune system. Biochem J 2011; 441: 339–346. 10.1042/BJ20111415 22007846

[bib23] Strickson S, Emmerich CH, Goh ETH, Zhang J, Kelsall IR, Macartney T et al. Roles of the TRAF6 and Pellino E3 ligases in MyD88 and RANKL signaling. Proc Natl Acad Sci USA 2017; 10.1073/pnas.1702367114.. 10.1073/pnas.1702367114 PMC5410814 28404732

[bib24] Rothwarf DM, Zandi E, Natoli G, Karin M. IKK-γ is an essential regulatory subunit of the IκB kinase complex. Nature 1998; 395: 297–300. 9751060 10.1038/26261

[bib25] Yamaoka S, Courtois G, Bessia C, Whiteside ST, Weil R, Agou F et al. Complementation cloning of NEMO, a component of the IκB kinase complex essential for NF-κB activation. Cell 1998; 93: 1231–1240. 9657155 10.1016/s0092-8674(00)81466-x

[bib26] Ea C-K, Deng L, Xia Z-P, Pineda G, Chen ZJ. Activation of IKK by TNFα requires site-specific ubiquitination of RIP1 and polyubiquitin binding by NEMO. Mol Cell 2006; 22: 245–257. 16603398 10.1016/j.molcel.2006.03.026

[bib27] Wu C-J, Conze DB, Li T, Srinivasula SM, Ashwell JD. Sensing of Lys 63-linked polyubiquitination by NEMO is a key event in NF-κB activation [corrected]. Nat Cell Biol 2006; 8: 398–406. 16547522 10.1038/ncb1384

[bib28] Hubeau M, Ngadjeua F, Puel A, Israel L, Feinberg J, Chrabieh M et al. New mechanism of X-linked anhidrotic ectodermal dysplasia with immunodeficiency: impairment of ubiquitin binding despite normal folding of NEMO protein. Blood 2011; 118: 926–935. 21622647 10.1182/blood-2010-10-315234 PMC3251327

[bib29] Döffinger R, Smahi A, Bessia C, Geissmann F, Feinberg J, Durandy A et al. X-linked anhidrotic ectodermal dysplasia with immunodeficiency is caused by impaired NF-κB signaling. Nat Genet 2001; 27: 277–285. 11242109 10.1038/85837

[bib30] Kirisako T, Kamei K, Murata S, Kato M, Fukumoto H, Kanie M et al. A ubiquitin ligase complex assembles linear polyubiquitin chains. EMBO J 2006; 25: 4877–4887. 17006537 10.1038/sj.emboj.7601360 PMC1618115

[bib31] Gerlach B, Cordier SM, Schmukle AC, Emmerich CH, Rieser E, Haas TL et al. Linear ubiquitination prevents inflammation and regulates immune signalling. Nature 2011; 471: 591–596. 21455173 10.1038/nature09816

[bib32] Ikeda F, Deribe YL, Skånland SS, Stieglitz B, Grabbe C, Franz-Wachtel M et al. SHARPIN forms a linear ubiquitin ligase complex regulating NF-κB activity and apoptosis. Nature 2011; 471: 637–641. 21455181 10.1038/nature09814 PMC3085511

[bib33] Tokunaga F, Nakagawa T, Nakahara M, Saeki Y, Taniguchi M, Sakata S et al. SHARPIN is a component of the NF-κB-activating linear ubiquitin chain assembly complex. Nature 2011; 471: 633–636. 21455180 10.1038/nature09815

[bib34] Lo Y-C, Lin S-C, Rospigliosi CC, Conze DB, Wu C-J, Ashwell JD et al. Structural basis for recognition of diubiquitins by NEMO. Mol Cell 2009; 33: 602–615. 19185524 10.1016/j.molcel.2009.01.012 PMC2749619

[bib35] Rahighi S, Ikeda F, Kawasaki M, Akutsu M, Suzuki N, Kato R et al. Specific recognition of linear ubiquitin chains by NEMO is important for NF-κB activation. Cell 2009; 136: 1098–1109. 19303852 10.1016/j.cell.2009.03.007

[bib36] Emmerich CH, Ordureau A, Strickson S, Arthur JSC, Pedrioli PGA, Komander D et al. Activation of the canonical IKK complex by K63/M1-linked hybrid ubiquitin chains. Proc Natl Acad Sci USA 2013; 110: 15247–15252. 23986494 10.1073/pnas.1314715110 PMC3780889

[bib37] Zhang J, Clark K, Lawrence T, Peggie MW, Cohen P. An unexpected twist to the activation of IKK*β*: TAK1 primes IKK*β* for activation by autophosphorylation. Biochem J 2014; 461: 531–537. 24911653 10.1042/BJ20140444 PMC4206954

[bib38] Tokunaga F, Sakata S, Saeki Y, Satomi Y, Kirisako T, Kamei K et al. Involvement of linear polyubiquitylation of NEMO in NF-κB activation. Nat Cell Biol 2009; 11: 123–132. 19136968 10.1038/ncb1821

[bib39] Kensche T, Tokunaga F, Ikeda F, Goto E, Iwai K, Dikic I. Analysis of nuclear factor-κB (NF-κB) essential modulator (NEMO) binding to linear and lysine-linked ubiquitin chains and its role in the activation of NF-κB. J Biol Chem 2012; 287: 23626–23634. 22605335 10.1074/jbc.M112.347195 PMC3390637

[bib40] Häcker H, Karin M. Regulation and function of IKK and IKK-related kinases. Sci STKE 2006; 2006: re13. 17047224 10.1126/stke.3572006re13

[bib41] Takaoka A, Yanai H, Kondo S, Duncan G, Negishi H, Mizutani T et al. Integral role of IRF-5 in the gene induction programme activated by toll-like receptors. Nature 2005; 434: 243–249. 15665823 10.1038/nature03308

[bib42] Lopez-Pelaez M, Lamont DJ, Peggie M, Shpiro N, Gray NS, Cohen P. Protein kinase IKK*β*-catalyzed phosphorylation of IRF5 at Ser462 induces its dimerization and nuclear translocation in myeloid cells. Proc Natl Acad Sci USA 2014; 111: 17432–17437. 25326418 10.1073/pnas.1418399111 PMC4267347

[bib43] Ren J, Chen X, Chen ZJ. IKK*β* is an IRF5 kinase that instigates inflammation. Proc Natl Acad Sci USA 2014; 111: 17438–17443. 25326420 10.1073/pnas.1418516111 PMC4267374

[bib44] Lang V, Symons A, Watton SJ, Janzen J, Soneji Y, Beinke S et al. ABIN-2 forms a ternary complex with TPL-2 and NF-κB1 p105 and is essential for TPL-2 protein stability. Mol Cell Biol 2004; 24: 5235–5248. 15169888 10.1128/MCB.24.12.5235-5248.2004 PMC419892

[bib45] Aoki M, Hamada F, Sugimoto T, Sumida S, Akiyama T, Toyoshima K. The human cot proto-oncogene encodes two protein serine/threonine kinases with different transforming activities by alternative initiation of translation. J Biol Chem 1993; 268: 22723–22732. 8226782

[bib46] Beinke S, Robinson MJ, Hugunin M, Ley SC. Lipopolysaccharide activation of the TPL-2/MEK/extracellular signal-regulated kinase mitogen-activated protein kinase cascade is regulated by IκB kinase-induced proteolysis of NF-κB1 p105. Mol Cell Biol 2004; 24: 9658–9667. 15485931 10.1128/MCB.24.21.9658-9667.2004 PMC522219

[bib47] Waterfield M, Jin W, Reiley W, Zhang M, Sun S-C. IκB kinase is an essential component of the Tpl2 signaling pathway. Mol Cell Biol 2004; 24: 6040–6048. 15199157 10.1128/MCB.24.13.6040-6048.2004 PMC480897

[bib48] Ben-Addi A, Mambole-Dema A, Brender C, Martin SR, Janzen J, Kjaer S et al. I B kinase-induced interaction of TPL-2 kinase with 14-3-3 is essential for toll-like receptor activation of ERK-1 and -2 MAP kinases. Proc Natl Acad Sci USA 2014; 111: E2394–E2403. 24912162 10.1073/pnas.1320440111 PMC4060680

[bib49] Pattison MJ, Mitchell O, Flynn HR, Chen C-S, Yang H-T, Ben-Addi H et al. TLR and TNF-R1 activation of the MKK3/MKK6-p38α axis in macrophages is mediated by TPL-2 kinase. Biochem J 2016; 473: 2845–2861. 27402796 10.1042/BCJ20160502 PMC5095906

[bib50] Arthur JSC, Ley SC. Mitogen-activated protein kinases in innate immunity. Nat Rev Immunol 2013; 13: 679–692. 23954936 10.1038/nri3495

[bib51] Heyninck K, Kreike MM, Beyaert R. Structure–function analysis of the A20-binding inhibitor of NF-κB activation, ABIN-1. FEBS Lett 2003; 536: 135–140. 12586352 10.1016/s0014-5793(03)00041-3

[bib52] Lork M, Verhelst K, Beyaert R. CYLD, A20 and OTULIN deubiquitinases in inflammation and cell death, so similar yet so different. Cell Death Differ 2017; e-pub ahead of print 31 March 2017; doi: 10.1038/cdd.2017.46. 10.1038/cdd.2017.46 PMC5520167 28362430

[bib53] Haas TL, Emmerich CH, Gerlach B, Schmukle AC, Cordier SM, Rieser E et al. Recruitment of the linear ubiquitin chain assembly complex stabilizes the TNF-R1 signaling complex and is required for TNF-mediated gene induction. Mol Cell 2009; 36: 831–844. 20005846 10.1016/j.molcel.2009.10.013

[bib54] Sato Y, Fujita H, Yoshikawa A, Yamashita M, Yamagata A, Kaiser SE et al. Specific recognition of linear ubiquitin chains by the Npl4 zinc finger (NZF) domain of the HOIL-1L subunit of the linear ubiquitin chain assembly complex. Proc Natl Acad Sci USA 2011; 108: 20520–20525. 22139374 10.1073/pnas.1109088108 PMC3251058

[bib55] Emmerich CH, Bakshi S, Kelsall IR, Ortiz-Guerrero J, Shpiro N, Cohen P. Lys63/Met1-hybrid ubiquitin chains are commonly formed during the activation of innate immune signalling. Biochem Biophys Res Commun 2016; 474: 452–461. 27133719 10.1016/j.bbrc.2016.04.141 PMC4880150

[bib56] Fiil BK, Damgaard RB, Wagner SA, Keusekotten K, Fritsch M, Bekker-Jensen S et al. OTULIN restricts Met1-linked ubiquitination to control innate immune signaling. Mol Cell 2013; 50: 818–830. 23806334 10.1016/j.molcel.2013.06.004 PMC4194427

[bib57] Nanda SK, Venigalla RKC, Ordureau A, Patterson-Kane JC, Powell DW, Toth R et al. Polyubiquitin binding to ABIN1 is required to prevent autoimmunity. J Exp Med 2011; 208: 1215–1228. 21606507 10.1084/jem.20102177 PMC3173241

[bib58] Nanda SK, Lopez-Pelaez M, Arthur JSC, Marchesi F, Cohen P. Suppression of IRAK1 or IRAK4 catalytic activity, but not type 1 IFN signaling, prevents lupus nephritis in mice expressing a ubiquitin binding–defective mutant of ABIN1. J Immunol 2016; 197: 4266–4273. 27807192 10.4049/jimmunol.1600788 PMC5114882

[bib59] Skaug B, Chen J, Du F, He J, Ma A, Chen ZJ. Direct, noncatalytic mechanism of IKK inhibition by A20. Mol Cell 2011; 44: 559–571. 22099304 10.1016/j.molcel.2011.09.015 PMC3237303

[bib60] Bosanac I, Wertz IE, Pan B, Yu C, Kusam S, Lam C et al. Ubiquitin binding to A20 ZnF4 is required for modulation of NF-κB signaling. Mol Cell 2010; 40: 548–557. 21095585 10.1016/j.molcel.2010.10.009

[bib61] Tokunaga F, Nishimasu H, Ishitani R, Goto E, Noguchi T, Mio K et al. Specific recognition of linear polyubiquitin by A20 zinc finger 7 is involved in NF-κB regulation. EMBO J 2012; 31: 3856–3870. 23032187 10.1038/emboj.2012.241 PMC3463848

[bib62] Verhelst K, Carpentier I, Kreike M, Meloni L, Verstrepen L, Kensche T et al. A20 inhibits LUBAC-mediated NF-κB activation by binding linear polyubiquitin chains via its zinc finger 7. EMBO J 2012; 31: 3845–3855. 23032186 10.1038/emboj.2012.240 PMC3463847

[bib63] Heyninck K, De Valck D, Vanden Berghe W, Van Criekinge W, Contreras R, Fiers W et al. The zinc finger protein A20 inhibits TNF-induced NF-κB-dependent gene expression by interfering with an RIP- or TRAF2-mediated transactivation signal and directly binds to a novel NF-κB-inhibiting protein ABIN. J Cell Biol 1999; 145: 1471–1482. 10385526 10.1083/jcb.145.7.1471 PMC2133159

[bib64] Coornaert B, Carpentier I, Beyaert R. A20: central gatekeeper in inflammation and immunity. J Biol Chem 2009; 284: 8217–8221. 19008218 10.1074/jbc.R800032200 PMC2659177

[bib65] Dong G, Chanudet E, Zeng N, Appert A, Chen Y-W, Au W-Y et al. A20, ABIN-1/2, and CARD11 mutations and their prognostic value in gastrointestinal diffuse large B-cell lymphoma. Clin Cancer Res 2011; 17: 1440–1451. 21266526 10.1158/1078-0432.CCR-10-1859

[bib66] Wertz IE, Newton K, Seshasayee D, Kusam S, Lam C, Zhang J et al. Phosphorylation and linear ubiquitin direct A20 inhibition of inflammation. Nature 2015; 528: 370–375. 26649818 10.1038/nature16165

[bib67] De A, Dainichi T, Rathinam CV, Ghosh S. The deubiquitinase activity of A20 is dispensable for NF-κB signaling. EMBO Rep 2014; 15: 775–783. 24878851 10.15252/embr.201338305 PMC4196981

